# 2-Isopropyl-5-methyl­cyclo­hexyl diphenyl­phospho­namidate

**DOI:** 10.1107/S1600536811010580

**Published:** 2011-03-31

**Authors:** Fan-Jie Meng, Chang-Qiu Zhao

**Affiliations:** a College of Chemistry and Chemical Engineering, Liaocheng University, Shandong 252059, People’s Republic of China

## Abstract

In the title compound, C_22_H_30_NO_2_P, the P atom has an irregular tetra­hedral geometry. In the crystal, mol­ecules are connected by N—H⋯O hydrogen-bonding inter­actions, giving rise to a chain along the *b* axis. The phenyl ring of the anilino group is twisted by 77.40 (16)° relative to the other phenyl ring. The absolute configuration of phospho­rus is *S*_p_.

## Related literature

For applications of chiral phosphinoyl­imines, see: Benamer *et al.* (2010 ▶). For related structures, see: Balakrishna *et al.* (2001 ▶). For the use of chiral organo­phospho­rus compounds in metal-catalyzed and organocatalytic reactions, see: Steinberg (1950 ▶).
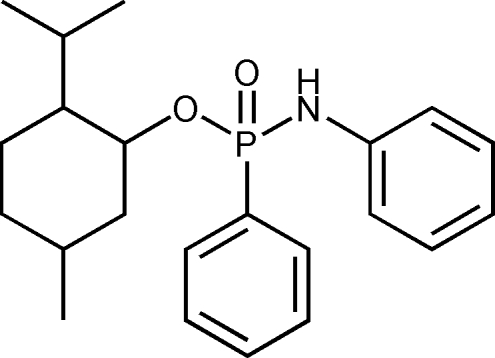


## Experimental

### 

#### Crystal data

C_22_H_30_NO_2_P
*M* *_r_* = 371.44
Monoclinic, 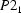

*a* = 8.6934 (8) Å
*b* = 5.4716 (5) Å
*c* = 22.100 (2) Å
β = 101.006 (1)°
*V* = 1031.90 (17) Å^3^
*Z* = 2
Mo *K*α radiation
μ = 0.15 mm^−1^
*T* = 298 K
0.45 × 0.36 × 0.17 mm

#### Data collection

Siemens SMART CCD area-detector diffractometer
Absorption correction: multi-scan (*SADABS*; Sheldrick, 1996 ▶) *T*_min_ = 0.936, *T*_max_ = 0.975
5183 measured reflections
3589 independent reflections
2814 reflections with *I* > 2σ(*I*)
*R*_int_ = 0.021

#### Refinement

*R*[*F* ^2^ > 2σ(*F* ^2^)] = 0.045
*wR*(*F* ^2^) = 0.104
*S* = 1.09
3589 reflections
238 parameters
1 restraint
H-atom parameters constrained
Δρ_max_ = 0.15 e Å^−3^
Δρ_min_ = −0.25 e Å^−3^
Absolute structure: Flack (1983 ▶), 1550 Friedel pairs
Flack parameter: −0.06 (12)

### 

Data collection: *SMART* (Siemens, 1996 ▶); cell refinement: *SAINT* (Siemens, 1996 ▶); data reduction: *SAINT*; program(s) used to solve structure: *SHELXS97* (Sheldrick, 2008 ▶); program(s) used to refine structure: *SHELXL97* (Sheldrick, 2008 ▶); molecular graphics: *SHELXTL* (Sheldrick, 2008 ▶); software used to prepare material for publication: *SHELXTL*.

## Supplementary Material

Crystal structure: contains datablocks I, global. DOI: 10.1107/S1600536811010580/bq2285sup1.cif

Structure factors: contains datablocks I. DOI: 10.1107/S1600536811010580/bq2285Isup2.hkl

Additional supplementary materials:  crystallographic information; 3D view; checkCIF report

## Figures and Tables

**Table 1:** Hydrogen-bond geometry (Å, °)

*D*—H⋯*A*	*D*—H	H⋯*A*	*D*⋯*A*	*D*—H⋯*A*
N1—H6⋯O2^i^	0.86	2.24	3.053 (3)	157

Symmetry code: (i) 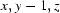.

**Fig. 1.**
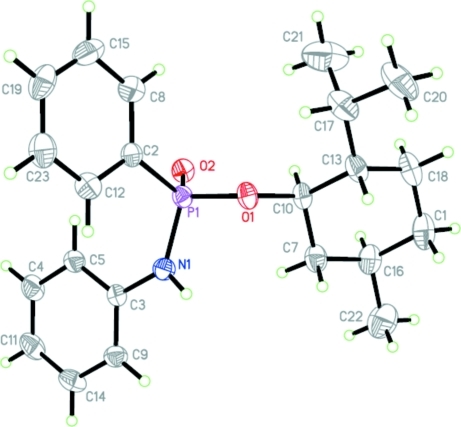
The molecular structure of (I). Displacement ellipsoids are drawn at the 50% probability level.

**Fig. 2.**
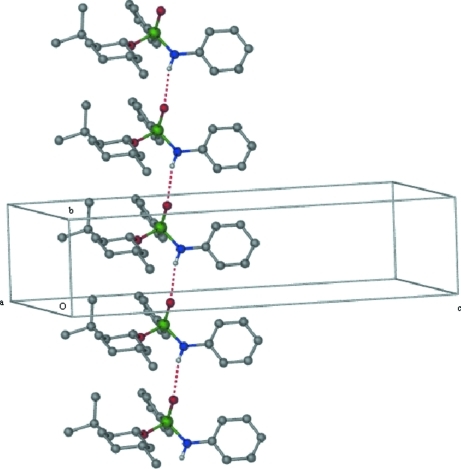
The one–dimensional chain of (I), linked by N—H···O hydrogen bonds.

## supplementary crystallographic information

### Comment

The catalytic asymmetric synthesis of chiral organophosphorus compounds has attracted considerable attention in the past decades, for these compounds can serve as precursors of many biologically active molecules and play an important role in metal-catalyzed and organocatalytic reactions (Steinberg, 1950). The molecular structure of the P-chiral title compound, (I), is composed of 2-isopropyl-5-methylcyclohexyl phenylphosphinate core with phenylamine (Fig. 1.). The configuration of the central P atom is S. The four groups around the P atom form a irregular tetrahedron (Benamer *et al.*, 2010). The torsion angles of the O(2)–P(1)–N(1)–C(3) and O(1)–P(1)–N(1)–C(3) are -45.0 (3) Å and -170.0 (2) Å. In the crystal structure (Balakrishna *et al.*, 2001), intermolecular N—H···O hydrogen bonds connect molecules into a one-dimensional chain (Table 1. , Fig. 2.).

### Experimental

Carbon tetrachloride was added to a solution of 2-isopropyl-5-methylcyclohexyl phenylphosphinate dissolved in dry ether and phenylamine. The reaction mixture was stirred for 38 h at room temperature. After washing with water, the resulting solution was purified by preparative TLC on silica gel to afford optically pure product. Single crystals of the title compound suitable for x–ray diffraction were obtained by slow evaporation of ether solution.

### Refinement

All H atoms attached to C atoms were fixed geometrically and treated as riding with C—H = 0.93–0.98 Å, with *U*_iso_(H) = 1.5 *U*_eq_(methyl) and *U*_iso_(H) = 1.2 *U*_eq_(C) for all other H atoms.

### Crystal data

**Table:**

C_22_H_30_NO_2_P	*F*(000) = 400
*M**_r_* = 371.44	*D*_x_ = 1.195 Mg m^−^^3^
Monoclinic, *P*2_1_	Mo *K*α radiation, λ = 0.71073 Å
Hall symbol: P 2yb	Cell parameters from 1942 reflections
*a* = 8.6934 (8) Å	θ = 2.7–25.2°
*b* = 5.4716 (5) Å	µ = 0.15 mm^−^^1^
*c* = 22.100 (2) Å	*T* = 298 K
β = 101.006 (1)°	Block, colorless
*V* = 1031.90 (17) Å^3^	0.45 × 0.36 × 0.17 mm
*Z* = 2	

### Data collection

**Table:**

Siemens SMART CCD area-detector diffractometer	3589 independent reflections
Radiation source: fine-focus sealed tube	2814 reflections with *I* > 2σ(*I*)
graphite	*R*_int_ = 0.021
φ and ω scans	θ_max_ = 25.0°, θ_min_ = 2.7°
Absorption correction: multi-scan (*SADABS*; Sheldrick, 1996)	*h* = −10→10
*T*_min_ = 0.936, *T*_max_ = 0.975	*k* = −6→6
5183 measured reflections	*l* = −26→13

### Refinement

**Table:**

Refinement on *F*^2^	Secondary atom site location: difference Fourier map
Least-squares matrix: full	Hydrogen site location: inferred from neighbouring sites
*R*[*F*^2^ > 2σ(*F*^2^)] = 0.045	H-atom parameters constrained
*wR*(*F*^2^) = 0.104	*w* = 1/[σ^2^(*F*_o_^2^) + (0.0475*P*)^2^ + 0.0269*P*] where *P* = (*F*_o_^2^ + 2*F*_c_^2^)/3
*S* = 1.09	(Δ/σ)_max_ < 0.001
3589 reflections	Δρ_max_ = 0.15 e Å^−^^3^
238 parameters	Δρ_min_ = −0.25 e Å^−^^3^
1 restraint	Absolute structure: Flack (1983), 1550 Friedel pairs
Primary atom site location: structure-invariant direct methods	Flack parameter: −0.06 (12)

### Special details

**Table:**

Geometry. All e.s.d.'s (except the e.s.d. in the dihedral angle between two l.s. planes) are estimated using the full covariance matrix. The cell e.s.d.'s are taken into account individually in the estimation of e.s.d.'s in distances, angles and torsion angles; correlations between e.s.d.'s in cell parameters are only used when they are defined by crystal symmetry. An approximate (isotropic) treatment of cell e.s.d.'s is used for estimating e.s.d.'s involving l.s. planes.
Refinement. Refinement of *F*^2^ against ALL reflections. The weighted *R*-factor *wR* and goodness of fit *S* are based on *F*^2^, conventional *R*-factors *R* are based on *F*, with *F* set to zero for negative *F*^2^. The threshold expression of *F*^2^ > σ(*F*^2^) is used only for calculating *R*-factors(gt) *etc*. and is not relevant to the choice of reflections for refinement. *R*-factors based on *F*^2^ are statistically about twice as large as those based on *F*, and *R*- factors based on ALL data will be even larger.

### Fractional atomic coordinates and isotropic or equivalent isotropic displacement parameters (Å^2^)

**Table:**

	*x*	*y*	*z*	*U*_iso_*/*U*_eq_	
C1	−0.0896 (4)	0.6404 (9)	0.08739 (15)	0.0830 (12)	
H1A	−0.0743	0.4702	0.0778	0.100*	
H1B	−0.1840	0.6970	0.0602	0.100*	
P1	0.36057 (8)	0.79230 (14)	0.29338 (3)	0.03738 (19)	
C2	0.5704 (3)	0.8039 (6)	0.30983 (11)	0.0402 (6)	
O2	0.2888 (2)	1.0296 (3)	0.30075 (8)	0.0446 (5)	
C3	0.2897 (3)	0.5828 (5)	0.39742 (11)	0.0351 (6)	
O1	0.3227 (2)	0.6846 (4)	0.22586 (8)	0.0436 (5)	
C4	0.3333 (4)	0.7801 (7)	0.49563 (12)	0.0570 (8)	
H4	0.3754	0.9087	0.5210	0.068*	
C5	0.3513 (3)	0.7748 (7)	0.43486 (11)	0.0459 (7)	
H5	0.4046	0.9000	0.4193	0.055*	
N1	0.3040 (3)	0.5712 (4)	0.33461 (10)	0.0424 (6)	
H6	0.2807	0.4340	0.3161	0.051*	
C7	0.0423 (3)	0.6000 (7)	0.19730 (13)	0.0543 (8)	
H7A	0.0304	0.6289	0.2395	0.065*	
H7B	0.0660	0.4282	0.1934	0.065*	
C8	0.6462 (4)	0.9995 (6)	0.28841 (14)	0.0566 (9)	
H8	0.5884	1.1207	0.2648	0.068*	
C9	0.2115 (3)	0.3967 (6)	0.42092 (13)	0.0458 (8)	
H9	0.1710	0.2659	0.3960	0.055*	
C10	0.1771 (3)	0.7509 (6)	0.18402 (11)	0.0439 (8)	
H10	0.1549	0.9240	0.1899	0.053*	
C11	0.2538 (4)	0.5968 (7)	0.51885 (14)	0.0611 (9)	
H11	0.2408	0.6024	0.5596	0.073*	
C12	0.6582 (4)	0.6285 (6)	0.34548 (13)	0.0507 (8)	
H12	0.6085	0.4972	0.3604	0.061*	
C13	0.2020 (4)	0.7132 (6)	0.11828 (12)	0.0534 (9)	
H13	0.2114	0.5362	0.1132	0.064*	
C14	0.1938 (4)	0.4062 (7)	0.48197 (14)	0.0590 (9)	
H14	0.1406	0.2815	0.4979	0.071*	
C15	0.8088 (4)	1.0140 (7)	0.30229 (16)	0.0660 (10)	
H15	0.8597	1.1438	0.2873	0.079*	
C16	−0.1120 (4)	0.6599 (7)	0.15376 (15)	0.0641 (10)	
H16	−0.1400	0.8292	0.1613	0.077*	
C17	0.3526 (5)	0.8226 (9)	0.10465 (16)	0.0738 (11)	
H17	0.4384	0.7565	0.1356	0.089*	
C18	0.0482 (4)	0.7874 (9)	0.07536 (13)	0.0765 (10)	
H18A	0.0285	0.9595	0.0812	0.092*	
H18B	0.0590	0.7642	0.0329	0.092*	
C19	0.8948 (4)	0.8369 (7)	0.33812 (16)	0.0634 (11)	
H19	1.0035	0.8479	0.3477	0.076*	
C20	0.3841 (5)	0.7449 (10)	0.04193 (16)	0.1023 (16)	
H20A	0.3830	0.5697	0.0392	0.153*	
H20B	0.4847	0.8050	0.0370	0.153*	
H20C	0.3044	0.8109	0.0100	0.153*	
C21	0.3603 (7)	1.0937 (9)	0.1111 (3)	0.1244 (19)	
H21A	0.2736	1.1659	0.0835	0.187*	
H21B	0.4567	1.1518	0.1013	0.187*	
H21C	0.3555	1.1379	0.1527	0.187*	
C22	−0.2440 (5)	0.4933 (9)	0.1665 (2)	0.0947 (14)	
H22A	−0.2187	0.3263	0.1595	0.142*	
H22B	−0.3403	0.5366	0.1396	0.142*	
H22C	−0.2556	0.5128	0.2086	0.142*	
C23	0.8201 (4)	0.6464 (7)	0.35934 (15)	0.0633 (10)	
H23	0.8783	0.5267	0.3834	0.076*	

### Atomic displacement parameters (Å^2^)

**Table:**

	*U*^11^	*U*^22^	*U*^33^	*U*^12^	*U*^13^	*U*^23^
C1	0.074 (3)	0.110 (3)	0.054 (2)	0.010 (3)	−0.0144 (19)	−0.006 (2)
P1	0.0433 (4)	0.0378 (4)	0.0310 (3)	0.0023 (4)	0.0073 (3)	−0.0027 (4)
C2	0.0466 (16)	0.0431 (15)	0.0316 (13)	−0.0011 (18)	0.0092 (11)	−0.0062 (16)
O2	0.0501 (13)	0.0402 (11)	0.0438 (11)	0.0053 (10)	0.0098 (9)	−0.0045 (10)
C3	0.0356 (15)	0.0374 (15)	0.0322 (14)	0.0056 (13)	0.0061 (11)	−0.0004 (13)
O1	0.0471 (11)	0.0513 (11)	0.0321 (10)	0.0098 (10)	0.0065 (8)	−0.0039 (8)
C4	0.083 (2)	0.0498 (17)	0.0370 (15)	0.002 (2)	0.0088 (14)	−0.0086 (18)
C5	0.0541 (17)	0.0452 (16)	0.0381 (15)	−0.0033 (18)	0.0077 (12)	−0.0003 (17)
N1	0.0543 (16)	0.0373 (13)	0.0365 (13)	−0.0036 (12)	0.0110 (11)	−0.0059 (11)
C7	0.052 (2)	0.069 (2)	0.0417 (17)	0.0033 (18)	0.0096 (14)	−0.0063 (17)
C8	0.057 (2)	0.056 (2)	0.056 (2)	−0.0012 (18)	0.0110 (16)	0.0024 (17)
C9	0.052 (2)	0.0386 (16)	0.0476 (18)	−0.0023 (14)	0.0106 (15)	0.0001 (14)
C10	0.0489 (17)	0.048 (2)	0.0322 (13)	0.0108 (16)	0.0015 (12)	−0.0048 (14)
C11	0.087 (3)	0.064 (2)	0.0363 (17)	0.011 (2)	0.0221 (17)	0.0024 (18)
C12	0.049 (2)	0.054 (2)	0.0504 (18)	0.0049 (16)	0.0124 (15)	0.0018 (16)
C13	0.070 (2)	0.058 (2)	0.0328 (15)	0.0075 (17)	0.0113 (15)	0.0003 (14)
C14	0.074 (3)	0.058 (2)	0.051 (2)	0.0003 (18)	0.0259 (18)	0.0130 (18)
C15	0.055 (2)	0.069 (2)	0.077 (2)	−0.021 (2)	0.0205 (19)	−0.011 (2)
C16	0.055 (2)	0.076 (2)	0.057 (2)	0.005 (2)	−0.0003 (16)	−0.0064 (18)
C17	0.081 (3)	0.087 (3)	0.058 (2)	0.010 (3)	0.0235 (18)	0.015 (2)
C18	0.090 (3)	0.098 (3)	0.0375 (17)	0.013 (3)	0.0002 (16)	0.006 (2)
C19	0.0388 (19)	0.085 (3)	0.066 (2)	0.002 (2)	0.0081 (16)	−0.025 (2)
C20	0.116 (3)	0.141 (5)	0.062 (2)	0.019 (3)	0.047 (2)	0.022 (3)
C21	0.155 (5)	0.084 (3)	0.153 (5)	−0.012 (4)	0.076 (4)	0.010 (3)
C22	0.060 (3)	0.114 (4)	0.106 (3)	−0.005 (3)	0.005 (2)	−0.012 (3)
C23	0.056 (2)	0.072 (3)	0.059 (2)	0.017 (2)	0.0045 (17)	−0.0031 (19)

### Geometric parameters (Å, °)

**Table:**

C1—C18	1.508 (5)	C11—C14	1.364 (5)
C1—C16	1.520 (4)	C11—H11	0.9300
C1—H1A	0.9700	C12—C23	1.386 (4)
C1—H1B	0.9700	C12—H12	0.9300
P1—O2	1.463 (2)	C13—C17	1.521 (5)
P1—O1	1.5795 (19)	C13—C18	1.539 (4)
P1—N1	1.645 (2)	C13—H13	0.9800
P1—C2	1.792 (3)	C14—H14	0.9300
C2—C12	1.376 (4)	C15—C19	1.378 (5)
C2—C8	1.386 (4)	C15—H15	0.9300
C3—C9	1.380 (4)	C16—C22	1.533 (5)
C3—C5	1.380 (4)	C16—H16	0.9800
C3—N1	1.419 (3)	C17—C21	1.490 (7)
O1—C10	1.463 (3)	C17—C20	1.524 (5)
C4—C11	1.372 (5)	C17—H17	0.9800
C4—C5	1.382 (4)	C18—H18A	0.9700
C4—H4	0.9300	C18—H18B	0.9700
C5—H5	0.9300	C19—C23	1.358 (5)
N1—H6	0.8600	C19—H19	0.9300
C7—C10	1.507 (4)	C20—H20A	0.9600
C7—C16	1.530 (4)	C20—H20B	0.9600
C7—H7A	0.9700	C20—H20C	0.9600
C7—H7B	0.9700	C21—H21A	0.9600
C8—C15	1.390 (4)	C21—H21B	0.9600
C8—H8	0.9300	C21—H21C	0.9600
C9—C14	1.388 (4)	C22—H22A	0.9600
C9—H9	0.9300	C22—H22B	0.9600
C10—C13	1.523 (4)	C22—H22C	0.9600
C10—H10	0.9800	C23—H23	0.9300
			
C18—C1—C16	112.6 (3)	C17—C13—C18	117.0 (3)
C18—C1—H1A	109.1	C10—C13—C18	106.7 (2)
C16—C1—H1A	109.1	C17—C13—H13	105.8
C18—C1—H1B	109.1	C10—C13—H13	105.8
C16—C1—H1B	109.1	C18—C13—H13	105.8
H1A—C1—H1B	107.8	C11—C14—C9	120.6 (3)
O2—P1—O1	114.83 (11)	C11—C14—H14	119.7
O2—P1—N1	114.34 (11)	C9—C14—H14	119.7
O1—P1—N1	102.61 (11)	C19—C15—C8	120.3 (3)
O2—P1—C2	112.65 (14)	C19—C15—H15	119.9
O1—P1—C2	103.04 (11)	C8—C15—H15	119.9
N1—P1—C2	108.29 (14)	C1—C16—C7	109.5 (3)
C12—C2—C8	119.1 (3)	C1—C16—C22	112.0 (3)
C12—C2—P1	121.5 (2)	C7—C16—C22	110.7 (3)
C8—C2—P1	119.3 (2)	C1—C16—H16	108.2
C9—C3—C5	119.9 (2)	C7—C16—H16	108.2
C9—C3—N1	118.5 (3)	C22—C16—H16	108.2
C5—C3—N1	121.6 (3)	C21—C17—C20	110.6 (4)
C10—O1—P1	120.25 (16)	C21—C17—C13	113.4 (4)
C11—C4—C5	120.4 (3)	C20—C17—C13	112.4 (4)
C11—C4—H4	119.8	C21—C17—H17	106.6
C5—C4—H4	119.8	C20—C17—H17	106.6
C3—C5—C4	119.7 (3)	C13—C17—H17	106.6
C3—C5—H5	120.1	C1—C18—C13	112.1 (3)
C4—C5—H5	120.1	C1—C18—H18A	109.2
C3—N1—P1	126.9 (2)	C13—C18—H18A	109.2
C3—N1—H6	116.5	C1—C18—H18B	109.2
P1—N1—H6	116.5	C13—C18—H18B	109.2
C10—C7—C16	112.4 (3)	H18A—C18—H18B	107.9
C10—C7—H7A	109.1	C23—C19—C15	119.7 (3)
C16—C7—H7A	109.1	C23—C19—H19	120.2
C10—C7—H7B	109.1	C15—C19—H19	120.2
C16—C7—H7B	109.1	C17—C20—H20A	109.5
H7A—C7—H7B	107.9	C17—C20—H20B	109.5
C2—C8—C15	119.9 (3)	H20A—C20—H20B	109.5
C2—C8—H8	120.1	C17—C20—H20C	109.5
C15—C8—H8	120.1	H20A—C20—H20C	109.5
C3—C9—C14	119.5 (3)	H20B—C20—H20C	109.5
C3—C9—H9	120.2	C17—C21—H21A	109.5
C14—C9—H9	120.2	C17—C21—H21B	109.5
O1—C10—C7	110.6 (2)	H21A—C21—H21B	109.5
O1—C10—C13	107.8 (2)	C17—C21—H21C	109.5
C7—C10—C13	111.6 (2)	H21A—C21—H21C	109.5
O1—C10—H10	108.9	H21B—C21—H21C	109.5
C7—C10—H10	108.9	C16—C22—H22A	109.5
C13—C10—H10	108.9	C16—C22—H22B	109.5
C14—C11—C4	119.8 (3)	H22A—C22—H22B	109.5
C14—C11—H11	120.1	C16—C22—H22C	109.5
C4—C11—H11	120.1	H22A—C22—H22C	109.5
C2—C12—C23	120.4 (3)	H22B—C22—H22C	109.5
C2—C12—H12	119.8	C19—C23—C12	120.7 (3)
C23—C12—H12	119.8	C19—C23—H23	119.7
C17—C13—C10	114.8 (3)	C12—C23—H23	119.7
			
O2—P1—C2—C12	134.9 (2)	C5—C4—C11—C14	0.9 (5)
O1—P1—C2—C12	−100.8 (2)	C8—C2—C12—C23	−0.5 (4)
N1—P1—C2—C12	7.4 (3)	P1—C2—C12—C23	−177.7 (2)
O2—P1—C2—C8	−42.3 (3)	O1—C10—C13—C17	−47.9 (4)
O1—P1—C2—C8	82.0 (2)	C7—C10—C13—C17	−169.6 (3)
N1—P1—C2—C8	−169.8 (2)	O1—C10—C13—C18	−179.3 (3)
O2—P1—O1—C10	−27.3 (2)	C7—C10—C13—C18	59.1 (3)
N1—P1—O1—C10	97.4 (2)	C4—C11—C14—C9	−0.5 (5)
C2—P1—O1—C10	−150.1 (2)	C3—C9—C14—C11	−0.4 (5)
C9—C3—C5—C4	−0.4 (4)	C2—C8—C15—C19	−1.1 (5)
N1—C3—C5—C4	179.4 (3)	C18—C1—C16—C7	−52.1 (5)
C11—C4—C5—C3	−0.5 (5)	C18—C1—C16—C22	−175.3 (3)
C9—C3—N1—P1	167.3 (2)	C10—C7—C16—C1	53.0 (4)
C5—C3—N1—P1	−12.5 (4)	C10—C7—C16—C22	177.0 (3)
O2—P1—N1—C3	−45.0 (3)	C10—C13—C17—C21	−62.8 (5)
O1—P1—N1—C3	−170.0 (2)	C18—C13—C17—C21	63.4 (5)
C2—P1—N1—C3	81.5 (2)	C10—C13—C17—C20	170.8 (3)
C12—C2—C8—C15	1.0 (4)	C18—C13—C17—C20	−63.0 (5)
P1—C2—C8—C15	178.2 (2)	C16—C1—C18—C13	57.0 (5)
C5—C3—C9—C14	0.8 (4)	C17—C13—C18—C1	171.6 (3)
N1—C3—C9—C14	−179.0 (3)	C10—C13—C18—C1	−58.3 (4)
P1—O1—C10—C7	−81.0 (3)	C8—C15—C19—C23	0.7 (5)
P1—O1—C10—C13	156.7 (2)	C15—C19—C23—C12	−0.2 (5)
C16—C7—C10—O1	−178.9 (2)	C2—C12—C23—C19	0.1 (5)
C16—C7—C10—C13	−58.9 (3)		

### Hydrogen-bond geometry (Å, °)

**Table:**

*D*—H···*A*	*D*—H	H···*A*	*D*···*A*	*D*—H···*A*
N1—H6···O2^i^	0.86	2.24	3.053 (3)	157

Symmetry codes: (i) *x*, *y*−1, *z*.

## References

[bb1] Balakrishna, M. S., Abhyankar, R. M. & Walawalker, M. G. (2001). *Tetrahedron Lett.* **42**, 2733–2734.

[bb2] Benamer, M., Turcaud, S. & Royer, J. (2010). *Tetrahedron Lett.* **51**, 645–648.

[bb3] Flack, H. D. (1983). *Acta Cryst.* A**39**, 876–881.

[bb4] Sheldrick, G. M. (1996). *SADABS* University of Göttingen, Germany.

[bb5] Sheldrick, G. M. (2008). *Acta Cryst.* A**64**, 112–122. 10.1107/S0108767307043930 18156677

[bb6] Siemens (1996). *SMART* and *SAINT* Siemens Analytical X-ray Instruments Inc., Madison, Wisconsin, USA.

[bb7] Steinberg, G. M. (1950). *J. Org. Chem.* **15**, 637–647.

